# Advancing our Understanding of Heat Wave Criteria and Associated Health Impacts to Improve Heat Wave Alerts in Developing Country Settings

**DOI:** 10.3390/ijerph16122089

**Published:** 2019-06-13

**Authors:** Amruta Nori-Sarma, Tarik Benmarhnia, Ajit Rajiva, Gulrez Shah Azhar, Prakash Gupta, Mangesh S. Pednekar, Michelle L. Bell

**Affiliations:** 1 Yale School of Forestry & Environmental Studies, New Haven, CT 06511, USA; Ajit.rajiva@gmail.com (A.R.); michelle.bell@yale.edu (M.L.B.); 2 Department of Family Medicine and Public Health and Scripps Institute of Oceanography, University of California at San Diego, La Jolla, CA 92093, USA; tbenmarhnia@ucsd.edu; 3 Pardee RAND Graduate School, Santa Monica, CA 90401, USA; gazhar@rand.org; 4 Healis-Sekhsaria Institute for Public Health, Navi Mumbai, Maharashtra 400 701, India; pcgupta@healis.org (P.G.); pednekarm@healis.org (M.S.P.)

**Keywords:** climate change, extreme temperature events, heat waves, human health, mortality, PSM, temperature-mortality relationships

## Abstract

Health effects of heat waves with high baseline temperatures in areas such as India remain a critical research gap. In these regions, extreme temperatures may affect the underlying population’s adaptive capacity; heat wave alerts should be optimized to avoid continuous high alert status and enhance constrained resources, especially under a changing climate. Data from registrars and meteorological departments were collected for four communities in Northwestern India. Propensity Score Matching (PSM) was used to obtain the relative risk of mortality and number of attributable deaths (i.e., absolute risk which incorporates the number of heat wave days) under a variety of heat wave definitions (*n* = 13) incorporating duration and intensity. Heat waves’ timing in season was also assessed for potential effect modification. Relative risk of heat waves (risk of mortality comparing heat wave days to matched non-heat wave days) varied by heat wave definition and ranged from 1.28 [95% Confidence Interval: 1.11–1.46] in Churu (utilizing the 95th percentile of temperature for at least two consecutive days) to 1.03 [95% CI: 0.87–1.23] in Idar and Himmatnagar (utilizing the 95th percentile of temperature for at least four consecutive days). The data trended towards a higher risk for heat waves later in the season. Some heat wave definitions displayed similar attributable mortalities despite differences in the number of identified heat wave days. These findings provide opportunities to assess the “efficiency” (or number of days versus potential attributable health impacts) associated with alternative heat wave definitions. Findings on both effect modification and trade-offs between number of days identified as “heat wave” versus health effects provide tools for policy makers to determine the most important criteria for defining thresholds to trigger heat wave alerts.

## 1. Introduction

Interest in heat waves and extreme heat events has increased globally over the past few years, following high profile heat events in the US and Europe that significantly impacted human health. Previous analysis of the 1995 Chicago heat wave illustrated gaps in the methodologies for the comparison of health effects of heat waves across regions [1], which led to application of time-series methods for analyzing temperature-mortality relationships [2] and further time-series analysis of that heat wave period [3].

Previous literature on the relationship between heat waves and health has focused on developed country settings. For example, the French heat wave of 2003 was estimated to increase mortality up to 137% in Paris, with an excess of 14,800 deaths across six cities over a 19-day period [4,5]. Studies of the relationship between heat waves and health in the US have shown increased mortality risk of heat waves, modified by intensity and duration of those heat waves [6,7]. Additional European studies have examined the additional effect of many consecutive days of high temperature [8], showing that heat waves are associated with excesses of mortality above those expected from linear increments with increasing daily temperature.

Although previous literature focused on the health effects of heat waves occurring in northern latitudes, much less is known about the effect of heat waves on health in tropical climates and in Lower- and Middle-Income Countries (LMICs). Extreme temperatures have been frequently reported in developing countries such as India, where news reports of melting pavements during a heat wave in 2015 made international headlines [9]. The frequency of hot days and heat waves are predicted to increase across India under climate change [10,11]. However, few studies have attempted to assess the heat–health relationship in India across multiple cities using population-level data and systematic methods for analysis. One study of the May 2010 heat wave in Northwestern India found a 43% increase in mortalities in the city of Ahmedabad when compared with reference periods [12]. Another study found low levels of short-term mortality displacement in a subset of districts in New Delhi when compared with total mortalities in London and São Paolo [13]. There are many challenges to conducting multi-city regional studies in India, such as a lack of data availability for public health research [14]. 

Understanding the effects of heat waves on health is key to preparing vulnerable communities for heat waves and to estimating the health impacts of climate change, which is anticipated to increase the frequency, duration, and intensity of heat waves [11]. Further, populations in these regions may be particularly vulnerable to heat and less able to adapt to climate change [15]. Therefore, scientific evidence on the impact of extreme temperatures in urban environments in developing country settings is a critically important research gap, which our study aims to fill. We show how local policy makers can use a variety of relative temperature metrics and an understanding of the potential risks of varying periods of extreme temperature to establish and implement heat action plans (HAPs), which aim to minimize the health outcomes of periods of extreme temperatures. Understanding how heat waves affect health is also necessary to estimate how climate change will affect health in developing country settings, particularly as baseline temperatures rise and periods of extreme heat become more frequent, intense, and longer in duration.

One major complexity in the generation of HAPs arises because there exists no standard definition of a “heat wave”, either in scientific research or in the policy community. The health impacts of a specific definition of a heat wave or design of a heat wave alert system is likely to vary across populations and communities. The health effect estimates of heat waves may be sensitive to temperature thresholds, lag structures, and variability in temperature over time [6,7,8,16]. Several temperature indices (e.g., maximum temperature, minimum temperature, heat index, regional temperature thresholds based on the whole temperature record) as well as time periods exceeding the established threshold are used by local, state, and national governments to declare heat waves [17]. Further, the timing of the heat wave within the season may have an effect on the health outcome associated with that heat wave, a factor that should be considered within HAPs. Finally, the accuracy of temperature forecasts may affect the health impacts of HAPs [18]. 

Most previous health studies of heat waves focused on intensity and duration characteristics of the heat wave (HW). In this study, in addition to these characteristics of HW definitions, we explore if seasonality contributes to the health impact related to heat waves. Our hypothesis is that heat waves occurring later in season may be more deleterious to human health in resource-constrained regions such as northwestern India, which relies on monsoon rains post-hot season to replenish water tables. Specifically, we assess to what extent the timing of the HW within the corresponding HW season modifies the relationship between HW and mortality. Hot seasons intersperse with the Northeastern and Southwestern monsoon seasons in some coastal areas in India; for example, within our dataset, Mumbai falls into this category. Associated meteorological differences in these communities could lead to different seasonal effects of heat wave periods. Some earlier studies in other study areas found that the first HW of the hot season had higher health risks than later HWs [6,19], which could relate to adaptation of the population or vulnerable populations succumbing early in the season (mortality displacement effects). 

In addition, we propose an innovative analytical approach to assess the causal effect of HW on mortality through the use of propensity score matching (PSM). Propensity score techniques aim to achieve covariate balance in regards to measured time-varying covariates (i.e., confounders) [20]. This approach also allows one to clearly formulate the causal question of interest. In this paper, we apply an approach based on propensity score matching to estimate the effect of heat waves on mortality. 

We examined the effect of the following parameters on the risk of mortality among five cities in northwestern India: (1) HW Definition, incorporating both percentile thresholds and heat wave duration; and (2) HW Timing in Season, assessing whether the heat wave was the first, second, third, fourth, fifth, or later in season.

## 2. Materials and Methods

### 2.1. Data

Daily temperature data and other meteorological data were collected from five cities in India: Jaipur (Rajasthan), Churu (Rajasthan), Idar (Gujarat), Himmatnagar (Gujarat), and Mumbai (Maharashtra). These cities represent a variety of climate conditions experienced throughout the states of Maharashtra, Gujarat, and Rajasthan in northwestern India; the state of Rajasthan records some of the hottest temperatures within cities in India [21]. Meteorological data were obtained from the Indian Meteorological Department (IMD) through their Metadata center in Pune and supplemented with data from the National Oceanic and Atmospheric Administration (NOAA) Global Summary of the Day (GSOD) project. Because meteorological stations were not available for Idar and because the cities of Idar and Himmatnagar are close in proximity (approximately 40 km from city center to city center), Idar and Himmatnagar were combined into a single community for subsequent analysis. 

Although the Civil Registration Act of 1952 requires municipality-level birth and death registration for the purpose of property transfers, no central registry for mortality data for India exists for research purposes. We collected detailed registry data on daily mortalities from the relevant city-level registry offices through 2012. The data were available over different study periods for each community depending on the availability of data within the relevant local registry. The four communities and associated time frames included in our study period were Jaipur (2005–2012) and Churu (2003–2012) in Rajasthan; Idar and Himmatnagar (2008–2012) in Gujarat; and the Island City of Mumbai (2000–2012) in Maharashtra. Data from the Island City of Mumbai were collected from the local registry for persons ≥35 years of age; for other cities, data were collected for persons of all ages. These data, which do not provide details on cause of death, were collated into all-cause aggregated daily mortality data for each of the communities of interest. To our knowledge, this is the first such compilation of detailed registry date for multiple Indian communities for the purpose of epidemiologic analysis and public health [14]. Data collection was approved by the Yale University Human Subjects Committee (HSC# 1405014004).

### 2.2. Heat Wave Definitions

To understand the impact of different heat wave definitions on the association between HWs and mortality, a variety of heat wave definitions by temperature threshold (intensity) and number of consecutive days of exceeding the threshold (duration) were applied to each community, as seen in Table 1. Thresholds were based on each community separately. Under these heat wave definitions, Mumbai is highlighted as a coastal city which experiences a mid-season monsoon period. Other cities in the data set do not experience a mid-season monsoon period, and the resulting hot season is shorter in duration. 

A notable exception in the list of heat wave definitions is the “India Meteorological Department (IMD)” definition. Meteorologists within the IMD use community-specific parameters to define conditions for when a “heat wave” is declared based on the historical temperature record of a given location, with forecasts limited to the temperature today and tomorrow as opposed to predicting heat waves multiple days in advance. The IMD definition of a heat wave allows for a single day of high temperature to be declared as a “heat wave”, in addition to multiple consecutive days of high temperature [22]. This contrasts with more common definitions of a “heat wave” in which at least two consecutive days of high temperature are necessary for the days to be considered a heat wave [23]. We included the IMD definition of a heat wave in our analysis to improve the policy relevance, and illustrate how the IMD definition of a heat wave compares to other standards in the literature.

Another methodological issue in assessing the relationship between heat waves and health is whether the heat metric used is an absolute heat metric (i.e., the same heat metric across all cities, such as a given temperature or threshold for heatwaves) or a relative metric (i.e., a percentile of temperature based on each community’s temperature distribution). Both have been used in the literature [6,7]. These metrics (absolute versus relative) have different interpretations and implications with respect to adaptation, which is an important consideration for climate change studies and public health policy. Acclimatization can occur through physical adaptation, housing characteristics, or behavioral patterns (e.g., staying indoors, clothing choices, water consumption). With a high degree of acclimatization to weather, results of heat wave-health assessments would be similar across communities for relative temperature effects and different for absolute effects. Without a high degree of acclimatization, communities would have similar absolute effects and dissimilar relative effects. In our models, we utilized a relative threshold, based on a community’s long-term weather, to allow for regional acclimatization to temperatures normal for these communities and to increase the comparability across different communities with different long-term meteorological trends.

### 2.3. Propensity Score Matching

PSM was used to control for confounding factors including longer-term patterns (time trends), seasonal and cyclical variations, short-term systematic effects (calendar effects including day of the week, weekend versus weekday) and other short-term time varying confounders (meteorological variables, in this case, adjusted dew point temperature) [24,25].

In our context, the underlying causal question focuses on the estimated health outcome rate on heat waves days, had the heat wave days not occurred. In other words, the outcome rate of interest was compared using heat wave days and non-heat wave days that were similar for all measured background covariates (except the daily temperature that was used to define a heat wave day). In addition, this approach allowed an intermediate stage in which it was possible to check covariate balance and eventually exclude heat wave days that could not be matched to any non-heat wave (i.e., control) days and vice versa.

Ensuring that the distribution of observed baseline covariates is similar and balanced between exposed and unexposed subjects (referred to as exchangeability) is fundamental when making causal inferences [26]. To reduce the potential for confounding within this observational study, PSM was used to compare heat wave days according to the heat wave definitions provided in Table 1 with the most similar non-heat wave days based on available data on measured confounders. We estimated the propensity score for each day and community in the study period using a parametric method, logistic regression, to predict the probability for a day to be a HW. This prediction was based on covariates included in the PSM model to generate a model to identify matched non-HW days. In the final model, which was run separately for each community and HW definition, we included the following covariates: day of the week (DOW) (weekend versus weekday), month, year, and lag temperatures (lag 1 to lag 3) as follows:

(1) $$logit\;P^{c}\left[HW=1|X\right]=\beta_{0}^{c}+\;\beta_{1}^{c}\left(DOW\right)+\beta_{2}^{c}\left(month\right)+\;\beta_{3}^{c}\left(year\right)+\;\overset{3}{\underset{n=1}{\sum }}\beta_{4n}^{c}T_{lag_{n}}^{o,c}$$

We estimated for each day and community a propensity score that was used to match HW days to similar non-HW days. Visual comparisons of kernel density plots of the resulting propensity scores as well as the standardized mean differences of each covariate were used to judge whether the matching improved covariate balance and to select the number of “nearest neighbors” and caliper size. We thus used “nearest-neighbor” matching [27] to match HW days to similar non-HW days with a ratio of 1:1 and a caliper of 0.1. Using this approach, we matched HW days to their nearest non-HW day neighbor (with regards to the propensity score). The number of days that were unmatched and thus excluded from the analysis varied between 1.22% in Jaipur to 5.91% in Mumbai. 

### 2.4. Association between Heat Waves and Mortality

Utilizing these balanced heat wave and non-heat wave days, we estimated the causal effect of HWs on mortality using a Quasi-Poisson regression model, which allowed for overdispersion of mortality count data. In this model, a dummy variable for heat wave day (with non-heat wave day as the control group) served as the predictor variable. This model yielded the average ratio in observed mortality between HW days and non-HW days. We obtained a Risk Ratio (RR) for each HW definition. In the model, we considered lags 0–14 days as well as cumulative lags; results presented are for lag 0. 

We repeated the same analysis separately for each community and included the population by year as an offset in the model. We also conducted sensitivity analysis in regards to the propensity score estimation to assess whether covariates in the model should be specified as a quadratic or linear function using likelihood ratio tests. Finally, we kept covariates modeled as a linear function in our PSM models.

### 2.5. Timing in Season

Different areas of India experience different seasons depending on latitude as well as the presence of northeastern and southwestern monsoons. For this reason, a standard “summer” has not been established for the continent. We analyzed the impact of timing in season on the health effects of all heat wave definitions for each community. Using the distribution of HWs across the year, we categorized these heat waves based on whether they were the first through fifth (or higher order) heat wave of the season.

### 2.6. Attributable Deaths versus Relative Risk

For each heat wave definition and community, attributable deaths were calculated by estimating the attributable risk percent (the percent of mortalities attributable to heat waves in relation to all mortalities) from the relative risk estimates, and multiplying by the daily average expected mortalities for the community and the number of heat wave days to obtain the total number of attributable number of deaths per heat wave definition [28,29]. This is similar to some previous papers which have sought to characterize the aggregate mortalities that are attributable to heat waves to better understand current health impacts of heat waves as well as potential future impacts of extreme heat under alternative climate scenarios [30,31].

(2) $$D_{a}=N_{HW}^{c}/year\times \;D_{e}^{c}\times \;\frac{\left(RR_{HW}^{c}-1\right)}{RR_{HW}^{c}}$$

where $N_{HW}^{c}/year$ = Number of heat wave days per year, varies by HW definition *HW* and community *c*; $D_{e}^{c}$ = average number of expected mortalities for community *c* ((annual mortality rate * community population)/365.25 days); $RR_{HW}^{c}$ = community-specific heat wave relative risk.

The estimated attributable risk includes the amplitude of the effect of the HW (i.e., through the $RR_{HW}^{c}$) and its prevalence (number of days on which a HW occurred). 

## 3. Results

Table 2 shows the descriptive statistics for the data that were used to assess the relationship between heat waves (by HW definition) and health outcomes, for all communities in our data set. We further illustrate the underlying differences in the populations of each of the communities as well as the different climatic regions that are represented in the analysis. We standardized all analyses by population, number of years of data available, and generated unique heat wave thresholds by community to ensure that our analyses were locally relevant.

Table 3 provides a further detailed breakdown of characteristics of different heat waves, by definition, for each community. We provide information on the average daily maximum temperature by heat wave definition, the number of days that were declared as heat waves under each definition, and the timing of the earliest heat wave over the entire record for each heat wave definition. Table 3 illustrates that, although heat wave definitions become more stringent across temperature and duration and, as a result, fewer days per year are declared as heat waves, the timing in season of the earliest heat wave does not change by more than a month across the communities.

**Figure 1:**
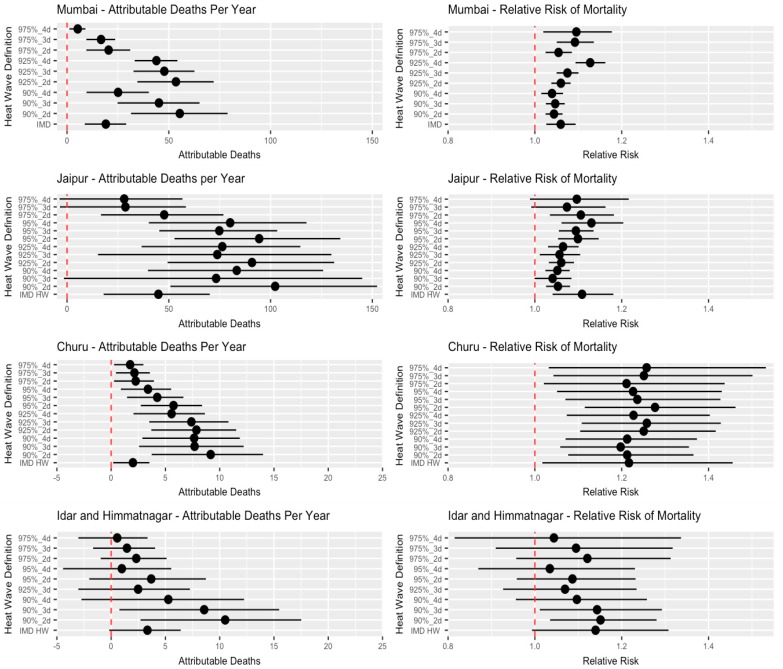
Annual average heat wave attributable deaths* and relative risk of mortality during a heat wave**, for all communities, by heat wave definitions (Where distinct heat wave periods occur). * Over whole study period for that community; see Table 1. ** A comparison of HW days to matched non-HW days. Note—Scale for attributable deaths is different by community.

Figure 1 shows the historical attributable deaths per year and relative risk of mortality associated with different heat wave definition criteria. Relative risk of mortality ranged from 1.28 [95% CI: 1.11–1.46] in Churu under the 95%_2d heat wave definition to 1.03 [95% CI: 0.87–1.23] in Idar and Himmatnagar under the 95%_4d definition. There is a wide spread in both the relative risk of heat waves and the attributable deaths associated with heat waves, indicating that both are largely dependent on the criteria used for declaring a heat wave. Further, Figure 1 shows the importance of going beyond the use of RR and examining the possibility of using health events to represent the impact of heat waves; the number of heat wave attributable deaths is an important driver of the variability of the HW-related burden in addition to relative risk. Some heat wave definitions were associated with a high relative risk; however, the absolute health burden is diminished because few days on record match those criteria.

**Figure 2:**
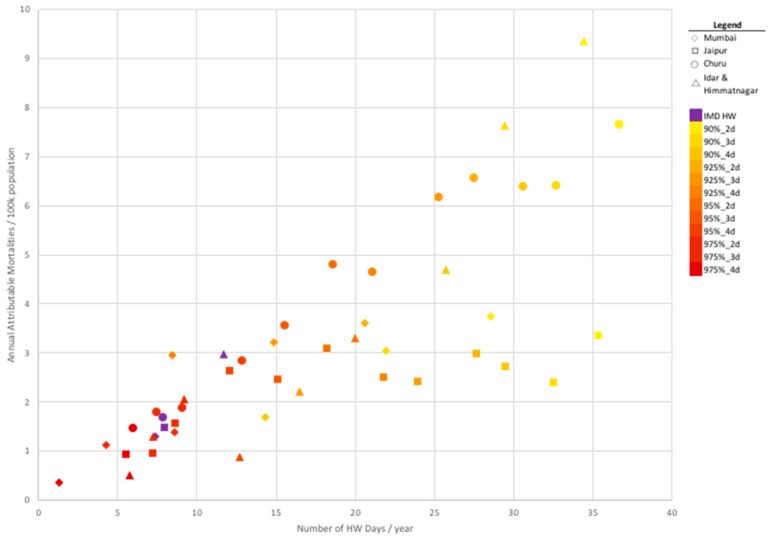
Number of heat wave days per year versus population-adjusted attributable deaths, by community and by heat wave definition.

To further investigate the relationship between aggregate mortalities and the prevalence of heat wave days, we applied a population weighting to the definition-specific attributable deaths estimates presented in Figure 1. Figure 2 shows the relationship between the number of days that fall into a particular heat wave definition and the annual population-weighted mortalities attributable to those heat wave days according to community. This shows that there are some heat wave definitions that are less stringent and occur often during the heat wave period; however, those definitions do not consistently correspond with the greatest number of attributable deaths annually. This may be an important “tradeoff” tool that can inform policy on heat wave alerts to maximize the public health impact while minimizing the resources expended during heat wave alerts; this may avoid saturating the public with heat wave warnings, which may lower responses to such alerts. 

**Figure 3:**
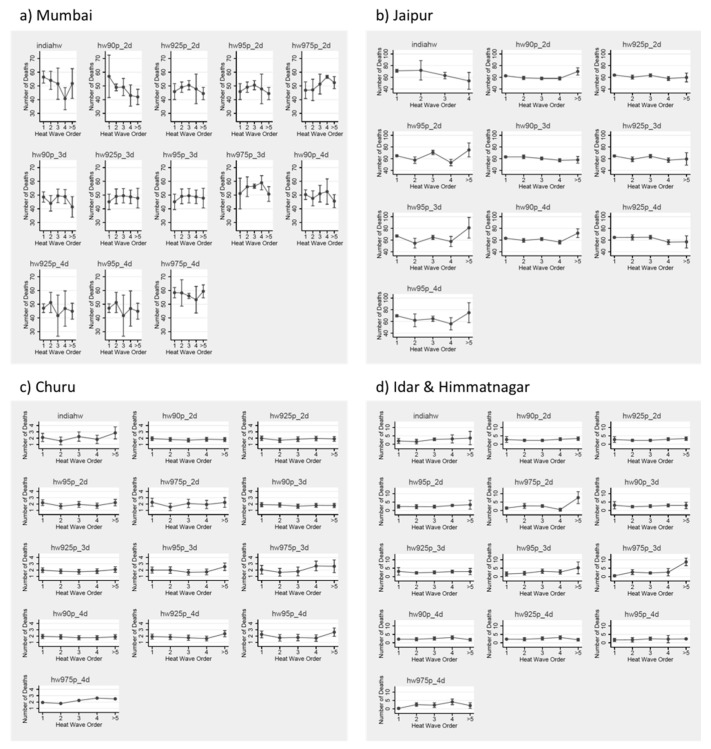
Number of deaths per each heat wave definition, by timing in season for all HW definitions (where distinct HW periods occur; definitions with identical outcomes removed) by community—(**a**) Mumbai, (**b**) Jaipur, (**c**) Churu, & (**d**) Idar & Himmatnagar

Figure 3 shows the within-season trend in the estimated relationship between the timing of heat waves and health. We assessed heat waves by order within the hot season: first, second, third, fourth, or greater than or equal to fifth. An important note is that this dataset occurs in a region where we do have several years of data with at least five or more heat wave periods, which is not common in the existing literature. Within this dataset, heat waves that occur later in the hot season tend to be more impactful on health. This holds true across different heat wave definitions, as well as in different communities. 

## 4. Discussion 

Because there is no standard definition of what constitutes a heat wave, implementation of heat wave early warning systems to minimize predicted health effects caused by heat waves can rely on data that examine the impact of a range of heat wave criteria (including potential effect modifiers) on past health effect estimates. This can help to generate locally relevant heat wave alert criteria that are tailored to maximize the reduction of health impacts in a particular region. For example, people may be less likely to respond to heat wave alerts depending on the frequency of those alerts; by contrast, the actual risk of an average HW may be lower if HWs are declared more frequently (i.e., if policy makers use a less stringent definition of a heat wave). Even if the same HW definition were to be used in the design of an alert program across multiple cities (e.g., a percentile of the city’s temperature distribution, which would be converted to a specific temperature value for implementation), health responses would differ depending on population characteristics. Further, some metrics or definitions of HWs may be more accurately predicted than others for HW warning systems. From a policy perspective, the actual choice of HW definition is a balance that involves tradeoffs of minimizing the health risks of heat waves and maximizing the efficient use of resources. This balance must come with an understanding that a lesser number of declared heat wave days will require less implementation of HAPs. 

The relationship between heat waves and health outcomes in this region is comparable to previous studies in other regions. Our results indicate that, even in areas with high baseline temperatures, adaptation to those high baseline temperatures is limited and the burden associated with extreme heat can be substantial. Future research focused on assessing the relationship between heat waves and health outcomes may identify a threshold temperature beyond which health effects of heat waves are consistent across geographic areas. 

Utilizing data on heat wave order, we found that heat waves that occur later in the season have a higher impact on health than those that occur earlier in the season. Previous studies have indicated that the first heat wave in the season tends to have the greatest health impacts [6,16], for several reasons: i) The most vulnerable populations succumb early in the hot season; ii) As the season progresses, the population implements adaptive behaviors (such as air conditioning usage) that offset the potential risk from heat waves. One study found that heat waves in the second half of the hot season had a greater health impact than heat waves in the first half of the hot season [21]; we provide evidence of higher heat wave effects in later order heat waves across multiple cities within a region that commonly experiences five or more heat wave periods during a single year. There are a few potential explanations for the later-in-season, high-impact heat waves that are specific to India: (i) Heat waves that occur after the monsoon season are likely to be more humid heat waves. It is clear and biologically plausibility that high-temperature, high-humidity periods are more deleterious to health than high-temperature, low-humidity periods; and (ii) There may be some other co-varying factor in areas of India that do not have mid-season monsoons, which might lead to more dangerous heat wave periods later in the season. It would be interesting to test such hypotheses in future studies. 

Our study is not without limitations. In our analysis, we used daily maximum temperature; however, the analysis could have used other meteorological characteristics, such as diurnal variation or daily minimum temperature. Future work could examine the relationships between other health-relevant meteorological characteristics and mortalities in the region, such as minimum and diurnal temperatures. We also utilized relative temperature thresholds for heat wave criteria, as opposed to absolute temperature metrics, to allow for acclimatization to high temperatures in these communities as well as for comparison across communities. Future studies could assess the relationship between absolute temperature metrics and heat wave-health outcomes to understand the level of acclimatization within the region. 

In terms of health data, we utilized data on aggregate, daily, all-cause mortality. For several of the cities in our study area (Himmatnagar, Jaipur), we do not have individual-level information and cannot assess important effect measure modifiers of the heat-mortality relationship [32,33], including demographic factors like age, gender, ethnicity, socioeconomic status (SES), behavior factors like smoking status and alcohol consumption, or cause of death such as chronic disease, cardiovascular disease, respiratory disease, among others. Future studies may utilize similar methods as presented above to assess these individual-level health characteristics as well as to understand sensitive subpopulations within these communities. 

A further limitation is that we estimated the IMD Heat Wave definition using publicly available data, which does not include the detailed datasets used by the IMD when issuing heat wave alerts. Although we believe that our estimation closely matches the criteria the IMD states in their handbook for issuing heat wave alerts, validation is difficult without access to IMD’s internal procedures. One major limitation to research in this region is the availability of data for use in public health studies. The quality of these data may be affected by different standards in reporting for registry data across different communities. 

Finally, we proposed a novel approach for time-series epidemiological studies that rely on the use of PSM. Advantages of such an approach include that it allows us to first check exchangeability between exposed (HW) and unexposed (non-HW) days before calculating the difference in a given health outcome using only one-dimensional parameter (the estimated PS). However, such an approach may still be prone to unmeasured confounding that can only be controlled to the extent that those unmeasured confounders are correlated with measured confounders, but such correlation is, by definition, impossible to check. 

## 5. Conclusions

Our study demonstrates the feasibility of utilizing locally relevant data from registries for public health-relevant work in India, an understudied region that currently experiences extreme temperatures. We further provide guidance for potential heat wave alert systems, which, by definition, must use a specific heat metric. As shown here, there are tradeoffs in the choice of an appropriate metric, including the resources expended. Longer periods of time are labeled as heat waves versus the numbers of mortalities which are attributable to these different types of heat waves, which could potentially be averted by implementation of an alert system.

India has begun the work of implementing heat wave alert systems to reduce the health impacts of heat waves [34]. Understanding the risk of heat waves to continue implementing heat action plans under a changing climate scenario may rely on critical examination of those high-risk, rare heat wave days that are highlighted in our results. One study conducted in Pakistan showed that knowledge and perception play a significant role in adaptation of populations to the health threat presented by heat waves; it also suggests that education on the health effects of heat waves will be a key component of successful implementation of health-protective measures during heat waves [35]. Climate change is expected to lead to more frequent heat waves of higher intensity and duration, which may lead to increases in the attributable health burden during those heat wave periods [11,15]. Health effect estimates from places such as India where high-intensity heat waves already occur may serve to inform the literature on health effects in other parts of the world that are anticipated to experience similar increases in temperature due to climate change. 

Future studies should aim to further develop our understanding of the local causal relationship between heat wave characteristics and health effects. Our study provides a highly useful framework for the value of registry data to build an understanding of the local heat wave-health relationships. Future studies could expand to other similar cities in India or other developing country settings to perform analogous research in resource-limited settings.

## Figures and Tables

**Table 1:** Heat Wave Definitions.

HW Label	Intensity: Temperature Threshold (Percentile of Temperature)	Duration: Minimum Duration Criteria (Days)
IMD HW	98th	>1
90%_2d	90th	>2
90%_3d	90th	>3
90%_4d	90th	>4
925%_2d	92.5th	>2
925%_3d	92.5th	>3
925%_4d	92.5th	>4
95%_2d	95th	>2
95%_3d	95th	>3
95%_4d	95th	>4
975%_2d	97.5th	>2
975%_3d	97.5th	>3
975%_4d	97.5th	>4

Note: To qualify for a HW (heat wave), each day must meet the minimum temperature threshold (for that community) on for the minimum number of consecutive days.

**Table 2:** Descriptive Statistics by Community.

Community	State	Census Population (millions) in Area (2011)	Study Period	Number of Deaths	Average Daily Maximum Temp (IQR) (°C)	Average Daily Dew Point Temp (IQR) (°C)
Island City, Mumbai	Maharashtra	1.5 (≥35 years)	2000–2012	216,635	32.5 (31, 34) ^1^	20.3 (16.9, 24) ^1^
Jaipur Municipal Area	Rajasthan	3.47	2005–2012	162,273	33.2 (29.5, 37.6) ^1^	13.4 (7, 21.2) ^1^
Churu Area	Rajasthan	0.12	2003–2012	5075	33.9 (29.3, 39.8) ^2^	N/A ^4^
Idar/Himmatnagar Area	Gujarat	0.11 (combined)	2006–2012	5682	34.2 (31, 37) ^3^	16.5 (11.2, 23.1) ^3^

^1^ Source: local monitor, ^2^ Source: nearest monitor in Bikaner (180 km from Churu), ^3^ Source: Himmatnagar monitor (30 km from Idar), ^4^ No nearby monitor available.

**Table 3:** Heat Wave Characteristics, by Definition.

	Average Temperature (°C)				Average # days/year				Earliest HW Start Date, Entire Record			
HW Label	Mumbai	Jaipur	Churu	Idar & H’nagar	Mumbai	Jaipur	Churu	Idar & H’nagar	Mumbai (Pre-, Post-Monsoon)	Jaipur	Churu	Idar & H’nagar
90%_2d	36.7	43.0	44.1	42.1	28.6	35.4	36.7	34.5	1/17, 9/30	4/14	4/8	3/14
90%_3d	36.7	43.1	44.2	42.2	22.0	32.6	32.7	29.5	2/19, 9/30	4/19	4/8	3/18
90%_4d	36.6	43.1	44.3	42.3	14.4	29.6	30.6	25.8	2/19, 10/4	4/22	4/8	4/13
925%_2d	37.0	43.1	44.5	42.1	20.6	27.7	27.4	34.5	1/23, 9/30	4/17	5/1	3/14
925%_3d	36.9	43.5	44.6	42.2	14.9	24.0	25.3	29.5	2/19, 9/30	4/25	5/1	3/18
925%_4d	37.1	43.3	44.7	42.3	8.5	21.9	21.1	25.8	2/28, 10/4	4/28	5/3	4/13
95%_2d	37.0	43.9	45.0	42.8	20.6	18.3	18.6	20.0	1/23, 9/30	4/17	5/4	3/14
95%_3d	37.0	44.1	45.2	42.9	14.9	15.1	15.6	16.5	2/19, 9/30	4/28	5/4	4/15
95%_4d	37.1	44.2	45.3	42.9	8.5	12.1	12.9	12.8	2/28, 10/4	5/1	5/4	4/15
975%_2d	37.6	44.7	45.9	43.5	8.6	8.7	9.1	9.3	2/8, 9/30	5/1	5/5	4/16
975%_3d	37.9	44.7	46.0	43.6	4.3	7.3	7.5	7.3	2/27, 10/6	5/5	5/5	4/16
975%_4d	37.4	44.8	46.2	43.6	1.3	5.6	6	5.8	3/10, 10/14	5/5	5/5	4/23
